# Differential functional immune recall across homologous and heterologous COVID-19 vaccination regimens

**DOI:** 10.3389/fimmu.2026.1921846

**Published:** 2026-08-06

**Authors:** Naim Che-Kamaruddin, Jefree Johari, Hasmawati Yahaya, Nurhafiza Zainal, Huy C. Nguyen, Robert D. Hontz, Andrew G. Letizia, Sazaly AbuBakar

**Affiliations:** 1 Tropical Infectious Diseases Research and Education Centre (TIDREC), Higher Institution Centre of Excellence (HICoE), Universiti Malaya, Kuala Lumpur, Malaysia; 2 Institute for Advanced Studies, Universiti Malaya, Kuala Lumpur, Malaysia; 3 Department of Medical Microbiology, Faculty of Medicine, Universiti Malaya, Kuala Lumpur, Malaysia; 4 U.S. Naval Medical Research Unit INDO PACIFIC, Singapore, Singapore

**Keywords:** cellular immune responses, COVID-19 booster vaccination, heterologous vaccination strategies, memory B cells, SARS-CoV-2 immunity

## Abstract

**Introduction:**

Understanding how different COVID-19 vaccine combinations shape long-term immunity is essential for improving durability and guiding booster strategies. Despite extensive characterization of neutralizing antibodies, long-term memory B and T cell responses after homologous and heterologous vaccination regimens remain poorly understood.

**Methods:**

In this study, peripheral blood mononuclear cells (PBMCs) from 50 individuals were analyzed 12 months after completion of a homologous two-dose primary COVID-19 vaccination series and had received a single booster dose (12-month follow-up). Participants initially received BNT162b2, ChAdOx1 nCoV-19, or CoronaVac as the primary vaccination series, followed by either a homologous booster or a heterologous BNT162b2 booster (ChAdOx1 nCoV-19/BNT162b2 and CoronaVac/BNT162b2). Unvaccinated individuals served as controls. B cell phenotypes were assessed using flow cytometry, while B and T cell recall responses were determined using ELISpot. IgG levels and cytokine/chemokine/growth factor profiles were evaluated using ELISA and Bio-Plex multiplex before and after *in vitro* stimulation.

**Results:**

The homologous BNT162b2 vaccination regimen exhibited significantly higher frequencies of memory B cells recognizing the ancestral (Wuhan strain-derived) SARS-CoV-2 Spike protein compared to the ChAdOx1 nCoV-19, CoronaVac, and unvaccinated controls. The CoronaVac/BNT162b2 vaccination regimen demonstrated significantly elevated RBD-specific IgG+ memory B cells and higher stimulated IgG levels, together with the highest IFN-γ-producing T cell responses. Homologous CoronaVac and CoronaVac/BNT162b2 vaccination regimens showed broader cytokine activation, including IL-6, IL-9, IL-15, TNF-α, and other cytokines. IL-6 levels were positively associated with memory B cell frequencies, suggesting a potential association with memory B cell differentiation and maintenance.

**Discussion:**

Different vaccination regimens were associated with distinct long-term cellular immune profiles under real-world conditions, with both homologous BNT162b2 and heterologous CoronaVac/BNT162b2 vaccination regimens maintaining robust memory B cell signatures and functional recall responses. The results highlight how priming combinations shape the durability and quality of immune memory, supporting the potential utility of mixed-platform booster strategies for sustaining long-term immunity.

## 1 Introduction

Persistent circulation of Severe Acute Respiratory Syndrome Coronavirus 2 (SARS-CoV-2) continues to pose significant public health challenges, resulting in a complex and highly variable immune landscape worldwide (1). A combination of natural infections and diverse vaccination strategies has created variable adaptive immune (1, 2). Despite the successful rollout of mass vaccination campaigns and high immunization rates, breakthrough infections and waning immunity remain critical concerns, particularly as new viral variants with immune-evasive properties emerge (3–6). The deployment of multiple vaccine platforms, including mRNA, viral vector, and inactivated vaccines along with both homologous (same-platform) and heterologous (mixed-platform) regimens, have further complicated our understanding of the long-term functional immune response (2, 7).

While antibody kinetics have been extensively studied, the sustained functionality of cellular immunity, particularly T cell and B cell-mediated responses, remains less characterized across studies (8–11). B cells are pivotal in SARS-CoV-2 immunity, not only through the production of neutralizing antibodies, but also by generating long-lived plasma cells and memory B cells that enable rapid and protective responses upon re-exposure to the virus (4, 9). Among these, switched memory B cells (CD27^+^ IgD^-^) are important mediators of long-term humoral immunity, particularly IgA and IgG responses (9). Heterologous vaccination regimens and hybrid immunity have been associated with broader and more robust B and T cell responses compared to homologous regimens, potentially enhancing long-term protection (4, 11). However, the precise mechanisms underlying these differences, especially regarding memory B cell subset dynamics, cytokine production, and their interplay with T cell responses remains insufficiently understood.

Malaysia represents a unique real-world setting in which multiple COVID-19 vaccine platforms, including mRNA (BNT162b2), adenoviral vector (ChAdOx1 nCoV-19), and inactivated virus (CoronaVac) were deployed concurrently (12), enabling direct comparison of diverse vaccination strategies. To address these knowledge gaps, this study aimed to evaluate the long-term (12-month) cellular immune responses in individuals who received different combinations of COVID-19 vaccines, including inactivated whole-virus vaccines. By comparing homologous and heterologous vaccination regimens, this study investigated the impact of vaccine platform and sequence on memory B cell phenotypes, functional recall capacity, and cytokine response profiles. These findings may help inform future vaccination strategies and improve understanding of long-term cellular immune responses across diverse COVID-19 vaccination regimens among the general public as well as those receiving vaccines due to occupational health risk, such as health care workers and military personnel, who could have multiple options for vaccine strategies if a new human coronavirus were to emerge.

## 2 Materials and methods

### 2.1 Ethical clearance

This study was conducted in accordance with the Declaration of Helsinki, and the protocol was approved by the Universiti Malaya Medical Centre-Medical Research Ethics Committee (MREC-UMMC SID: 2021226-9886) and the U.S. Navy Human Research Protections Office (HRPO.NAMRU2.2021.0002) in compliance with all applicable federal regulations governing the protection of human subjects. All participants provided informed consent for inclusion before they participated in the study.

### 2.2 Study design and PBMCs collection

This cross-sectional study involved the collection of peripheral blood mononuclear cells (PBMCs) from participants previously enrolled in a longitudinal serological study investigating SARS-CoV-2 immune responses in the Greater Kuala Lumpur region (13, 14); this sub-study represents an analysis at the 12-month follow-up, defined as 12 months after completion of the initial homologous two-dose primary COVID-19 vaccination series and approximately 6–8 months after receipt of a single booster dose. Participants had initially received a homologous two-dose primary COVID-19 vaccination series with either BNT162b2 (15), ChAdOx1 nCoV-19 (16), or CoronaVac (17). Participants subsequently received a single booster dose approximately 3–6 months following completion of the primary vaccination series, consisting of either the same vaccine platform (homologous booster) or BNT162b2 (heterologous booster). Participants were selected based on availability and willingness to provide additional blood samples. No randomization was performed. Sample collection was conducted from August 2022 to May 2023, corresponding to the 12-month follow-up, with no additional vaccine doses received following booster administration prior to sampling. For consistency, the term “12-month follow-up” is used throughout the manuscript to refer to sampling performed at the 12 months after completion of the primary vaccination series and approximately 6–8 months after booster vaccination.

A total of 50 healthy adults aged 18 to 45 years were enrolled. All participants were afebrile, had no known active COVID-19 disease, had no known immunosuppressive conditions, were not receiving immunosuppressive medications, and were not pregnant at the time of sampling. Vaccination status was verified using official vaccination certificates issued through the national digital health platform (MySejahtera, Ministry of Health Malaysia), which records individual vaccination status and provides a verifiable digital certificate. Participants were not systematically screened for interim SARS-CoV-2 infections between vaccination and PBMCs sampling. Therefore, undocumented symptomatic or asymptomatic infections could not be excluded, and the analysis reflects real-world immune status rather than vaccine-only exposure.

**Figure 1:**
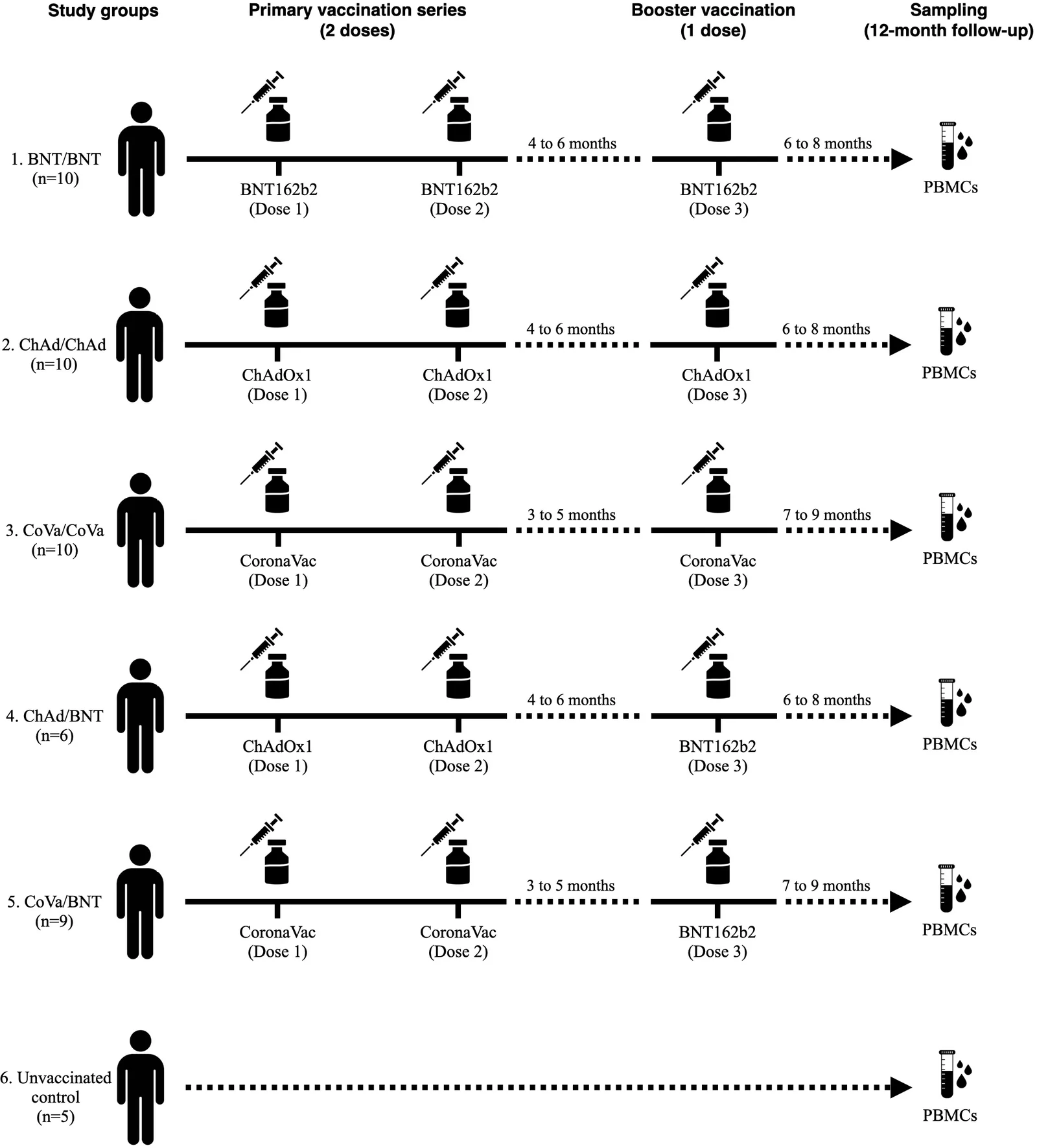
Study design and vaccination regimens. Participants received a homologous two-dose primary COVID-19 vaccination series with BNT162b2, ChAdOx1 nCoV-19, or CoronaVac, followed by either a homologous booster or a heterologous BNT162b2 booster administered approximately 3–6 months after completion of the primary series. PBMCs were collected at the 12-month follow-up after completion of the primary vaccination series (approximately 6–8 months post-booster). Unvaccinated individuals served as controls. Figure created using BioRender.com.

Participants were categorized into six study groups according to their COVID-19 vaccination regimens (**Figure 1**):

1. Homologous BNT162b2 (BNT/BNT, n=10).
2. Homologous ChAdOx1 nCoV-19 (ChAd/ChAd, n=10).
3. Homologous CoronaVac (CoVa/CoVa, n=10).
4. Heterologous ChAdOx1 nCoV-19 followed by BNT162b2 (ChAd/BNT, n=6).
5. Heterologous CoronaVac followed by BNT162b2 (CoVa/BNT, n=9).
6. Unvaccinated controls, defined as individuals without documented COVID-19 vaccination (n=5).

PBMCs were collected via venipuncture using BD Vacutainer^®^ CPT™ Mononuclear Cell Preparation Tubes (8 mL) tubes and isolated by density gradient centrifugation as previously described (18). The cells were cryopreserved in CellBanker 2^®^ cryomedium at –80 °C until analysis. Prior to analysis, PBMCs were thawed and washed in culture media for use in flow cytometry and ELISpot assays. Supernatants from both unstimulated and stimulated PBMCs cultures were collected for ELISA and Bio-Plex cytokine analysis.

### 2.3 Quantification of B cell population using flow cytometry

PBMCs were analyzed using flow cytometry to discern the frequency of B cell populations and their correlation with the memory response. Cell surface staining included the following markers and fluorochromes: CD19 (PE-CF594), CD20 (BV786), CD27 (PE-Cy7), CD24 (APC-R700), CD38 (BV650), IgD (BV421), and IgM (BB515), together with the viability dye 7-AAD. These markers enabled discrimination of mature B cell lineage (CD19^+^ CD20^+^), transitional B cells (CD38^dim/-^ CD24^+^), antibody-secreting cell (CD38^+^ CD24^-^ CD27^+^ CD20^-^), memory B cells, and naïve B cells based on CD27 and IgD expression patterns. SARS-CoV-2 spike protein (Spike) and RBD-specific SARS-CoV-2 B cells were identified using biotinylated Spike and RBD probes detected with BB515 Streptavidin (green) and BV421 Streptavidin (violet). This study’s gating strategy used two independently labeled streptavidin–Spike probes to minimize background signal and nonspecific binding. Only B cells that bound to both probes (dual-positive, also known as “Spike^++^”) were considered antigen-specific, thereby reducing potential nonspecific binding. IgM and IgG staining were used to distinguish the primary and secondary response. The complete flow cytometry gating strategy used to identify the analyzed B-cell subsets is provided in **Supplementary Figure 1**. Memory B cell subsets were defined according to previously published SARS-CoV-2 immune profiling studies (19, 20). A BD FASCLyric™ cell analyzer was used for this analysis and gating strategy was illustrated using FlowJo v.10.8 software.

### 2.4 Quantification of B and T cells recall response using ELISpot assay

The functionality of memory B and T cells was analyzed using PBMCs stimulated against SARS-CoV-2 antigens *in vitro*. Commercial ELISpot assays (Mabtech, Sweden) were used to quantify the production of RBD-specific IgG in memory B cells. The assays assessed interferon-gamma (IFN-γ) responses in memory T cells.

For B cell stimulation, PBMCs were activated using a polyclonal stimulus (R848 [Resiquimod] + IL-2; Mabtech, Sweden: 3850-4APW-R1-1) to induce differentiation on memory B cells into antibody-secreting cells. Subsequent ELISpot detection was performed using SARS-CoV-2 receptor-binding domain (RBD)-specific capture. Therefore, the assay quantified RBD-specific IgG-producing memory B cells.

For T cell stimulation, the following peptide pools were used: Wildtype S1 (SARS-CoV-2 S1, scanning pool, Mabtech, Sweden: 3629-1), Wildtype SNMO (SARS-CoV-2 SNMO, human peptide pool, Mabtech, Sweden: 3622-1), and Omicron (BA.1) S1 (SARS-CoV-2 Omicron BA.1, scanning pool, Mabtech, Sweden: 3636-1). A T cell positive control was included for each participant using anti-CD3 monoclonal antibody (Mabtech CD3-2), which serves as a mitogenic stimulus to confirm T cell functionality and assay performance.

Briefly, 2 x 10^5^ cells were cultured in RPMI supplemented with 10% fetal bovine serum and incubated with the peptides for 72 hours at 37 °C. Spot development was performed according to the manufacturer’s protocol (**Supplementary Figure 2**). Unstimulated controls for each sample served as an internal reference, enabling a comparative analysis of stimulated changes using each individual’s baseline as a reference point. Spot quantification was performed using an ImmunoSpot^®^ analyzer (CTL, USA), with results converted into spot-forming units per million cells (SFU/10^6^ cells) for standardization. Spot counts were calculated by taking the mean of triplicate wells (**Supplementary Figure 3**). The collected data were analyzed to determine the frequency of IgG-secreting memory B cells.

### 2.5 Quantification of SARS-CoV-2 S1-specific IgG levels using ELISA

SARS-CoV-2 S1-specific IgG levels in supernatants from unstimulated and stimulated PBMCs samples were measured using a commercial anti-SARS-CoV-2 QuantiVac ELISA (IgG) (EUROIMMUN, Lübeck, Germany) according to the manufacturer’s instructions. Quantitative determination of binding antibody units (BAU/mL) was obtained using the standards provided by the manufacturer with a positivity cut-off value of 35.2 BAU/mL. According to the manufacturer’s information, the sensitivity of the ELISA kit is 93.2%, and the specificity is 99.8%.

### 2.6 Quantification of cytokines, chemokines, and growth factors using Bio-Plex assay

PBMCs cytokine, chemokine, and growth factor levels were assessed using Bio-Plex multiplex magnetic bead-based human cytokine assay (Bio-Rad, CA, USA) using a 25-Plex Screening Panel. The Bio-Plex 200 Reader was used to measure median fluorescence intensities, and each sample underwent triplicate analysis. Data acquisition and analysis were performed using Bio-Plex Manager Software version 6.2. Standard curves for each cytokine were established using the manufacturer’s provided standards, according to the manufacturer’s instructions.

In this analysis, 25 immune signals involved in inflammatory responses and initiation of cellular immune signals were evaluated. These cytokines and chemokines included eotaxin (CCL11), fibroblast growth factor (FGF), granulocyte colony-stimulating factor (G-CSF), granulocyte-macrophage colony-stimulating factor (GM-CSF), interferon-γ (IFN-γ), interleukin 1 receptor antagonist (IL-1RA), IL-2, IL-4, IL-5, IL-6, IL-7, CXCL8 (also known as IL-8), IL-9, IL-10, IL-12 (p70), IL-13, IL-15, IL-17A, monocyte chemoattractant protein-1 (MCP-1, MCAF), macrophage inflammatory protein-1α (MIP-1α), MIP-1β, platelet-derived growth factor-BB, regulated upon activation, normal T-cell expressed and presumably secreted (RANTES), tumor necrosis factor-α (TNF-α), and vascular endothelial growth factor-A (VEGF).

### 2.7 Statistical analysis

Descriptive analyses of continuous variables were summarized as medians with interquartile ranges (IQR). For comparisons involving more than two independent groups, the Kruskal–Wallis test was used to evaluate differences in non-parametric continuous variables across vaccination regimen groups. Where appropriate, Mann–Whitney U tests were applied for pairwise comparisons, and Wilcoxon signed-rank tests were used for within-group (paired) analyses.

Box plots were used to visualize the distribution of B cell subpopulations, ELISpot-derived B and T cell responses, IgG levels, and cytokine, chemokine, and growth factor concentrations across vaccination regimens, displaying medians and interquartile ranges to illustrate differences in central tendency and variability. Principal Component Analysis (PCA) was performed on cytokine, chemokine, and growth factor datasets to reduce dimensionality and to visualize patterns of immune mediator responses among vaccination regimens. Prior to PCA, data were centered and scaled, and the proportion of variance explained by each principal component was reported.

All statistical analyses were conducted using R version 4.0.4 (R Foundation for Statistical Computing, Vienna, Austria). A two-sided *p*-values < 0.05 was considered statistically significant.

## 3 Results

### 3.1 Study participants

The median age of the 50 participants was 29.5 years (IQR: 25-36.8), with median ages across vaccination regimens ranging from 25 to 37.5 years. The cohort was predominantly female (66%), with variation in sex distribution across vaccination regimens. The median primary-to-booster interval ranged from 4 to 5 months across vaccination regimens, while the median booster-to-sampling interval ranged from 7 to 8 months (**Table 1**).

**Table 1:** Demographic characteristics of study participants according to COVID-19 vaccination regimen at the 12-month follow-up.

Study groups	Age, years median (IQR)	Female, n (%)	Primary-to-booster interval, months median (IQR)	Booster-to-sampling interval, months median (IQR)
1. BNT/BNT (n=10)	26.5 (25-34)	6 (60%)	5 (4-6)	7 (6-8)
2. ChAd/ChAd (n = 10)	27.5 (27-32)	5 (50%)	5 (4-6)	7 (6-8)
3. CoVa/CoVa (n = 10)	37.5 (35-38)	6 (60%)	4 (3-5)	8 (7-9)
4. ChAd/BNT (n=6)	25 (24-29)	5 (83.3%)	5 (4-6)	7 (6-8)
5. CoVa/BNT (n=9)	25 (23-27)	9 (100%)	4 (3-5)	8 (7-9)
6. Unvaccinated Control (n=5)	37 (35-40)	2 (40%)	Not applicable	Not applicable
**Overall (N = 50)**	**29.5 (25-36.8)**	**33 (66%)**	**5 (4-6)**	**7 (6-8)**

Age is presented as median (interquartile range, IQR). Sex distribution is shown as number (percentage) within each vaccination regimen study group. Primary-to-booster interval refers to the duration between completion of the two-dose primary vaccination series and administration of a booster dose. Booster-to-sampling interval refers to the duration between booster vaccination and PBMCs collection. Participants were categorized based on homologous or heterologous vaccination combinations, with an additional unvaccinated control group. Overall values summarize the combined study population (N = 50).

BNT, BNT162b2 vaccine; ChAd, ChAdOx1 nCoV-19 vaccine; CoVa, CoronaVac vaccine; “/” separates the two-dose primary vaccination series and a booster dose in chronological order.

Bold values represent the overall characteristics of all study participants combined (N = 50).

### 3.2 B cell subpopulation profile at the 12-month follow-up

**Figure 2:**
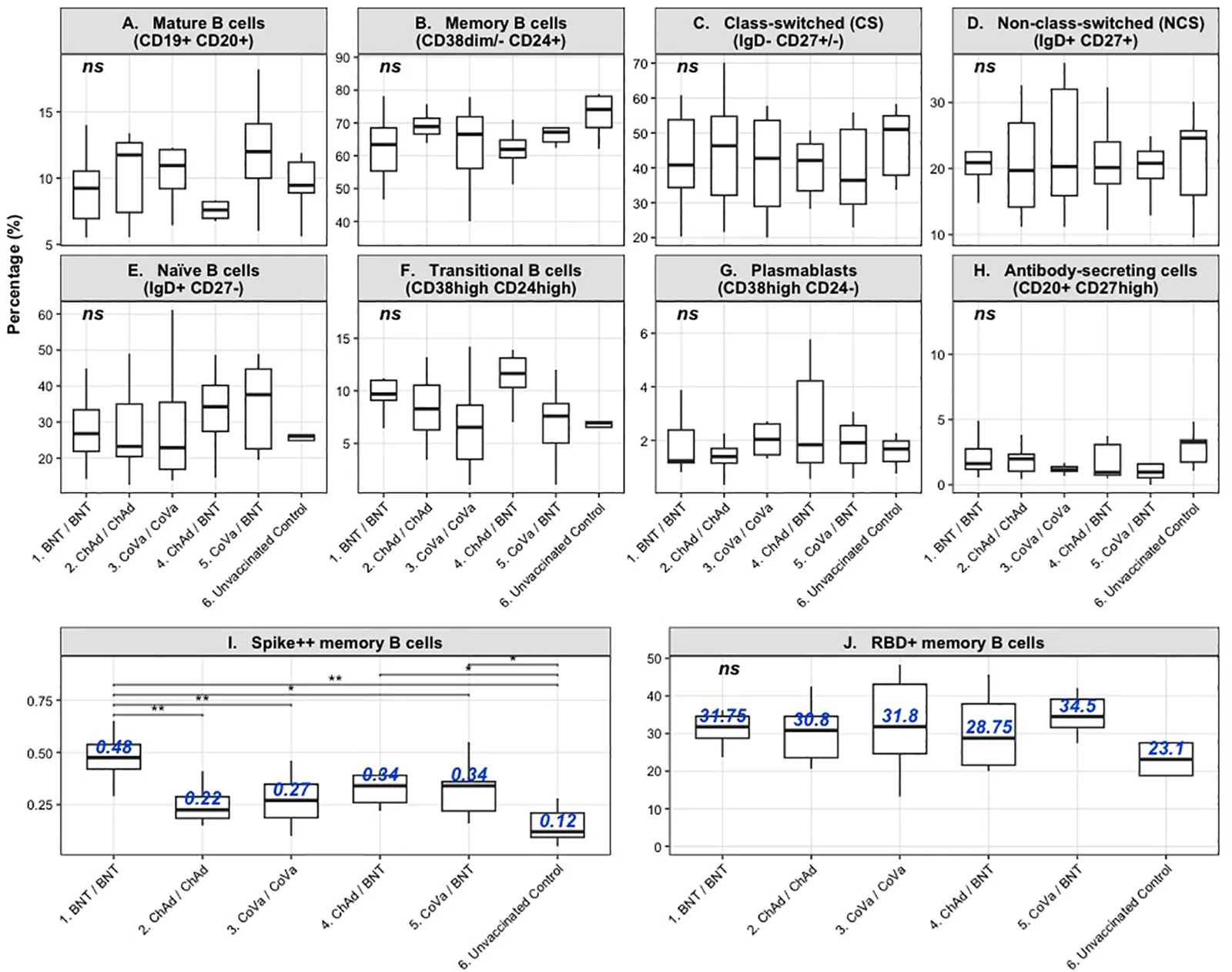
B-cell responses at the 12-month follow-up across different COVID-19 vaccination regimens. **(A–H)** present the frequencies of major B-cell subsets as percentages of the parental population defined by the gating strategy. **(I, J)** show the frequencies of Spike++ and RBD+ memory B cells, respectively. Box plots represent the median and interquartile range. Overall comparisons for **(A–H)** were performed using the Kruskal–Wallis test. Pairwise comparisons for **(I, J)** were performed using the Mann–Whitney U test. Significant differences are indicated as *p < 0.05 and **p < 0.01; “ns” denotes no significant difference. Blue numbers indicate the median values for Spike++ and RBD+ memory B cells. CS, class-switched; NCS, non-class-switched; ASCs, antibody-secreting cells; BNT, BNT162b2 vaccine; ChAd, ChAdOx1 nCoV-19 vaccine; CoVa, CoronaVac vaccine; “/” separates the two-dose primary vaccination series and the booster dose in chronological order.

At the 12-month follow-up, flow cytometric analysis of B cell subpopulations showed distinct response patterns among different vaccination regimens (**Figure 2**). The frequency of Spike^++^ B cell differed significantly across vaccination regimens. Individuals who received a homologous BNT162b2 vaccination regimen exhibited the highest frequency of Spike^++^ B cells compared to those who received homologous ChAdOX1 nCoV-19, homologous CoronaVac, heterologous ChAdOX1 nCoV-19/BNT162b2, heterologous CoronaVac/BNT162b2, and the unvaccinated control groups. In contrast, the RBD^+^ B cell frequencies were showed comparable across vaccination regimens (**Figure 2**).

Analysis of memory B cell subpopulations revealed a significant overall difference in the frequency of Spike^++^ memory B cell population across vaccination regimens (Kruskal-Wallis test). Subsequent pairwise comparisons using Mann-Whitney U tests showed that participants who received the homologous BNT162b2 vaccination regimen showed a significantly higher frequency of Spike^++^ memory B cells (median = 0.48%) compared to the homologous ChAdOx1 nCoV-19 (median = 0.22%, *p* < 0.005), homologous CoronaVac (median = 0.27%, *p* < 0.005), heterologous CoronaVac/BNT162b2 (median = 0.34%, *p* < 0.05), and the unvaccinated control groups (median = 0.12%, *p* < 0.005).

Both heterologous booster regimens (ChAdOX1 nCoV-19/BNT162b2 and CoronaVac/BNT162b2) also demonstrated significantly higher Spike^++^ memory B cell frequencies compared to the unvaccinated controls (*p* < 0.05). In contrast, although the percentage of the RBD^+^ memory B cell population varied across vaccination regimens (medians ranging from 23.1% to 34.5%), these differences were not statistically significant (**Figure 2**).

### 3.3 ELISpot analysis of B and T cell responses at the 12-month follow-up

**Figure 3:**
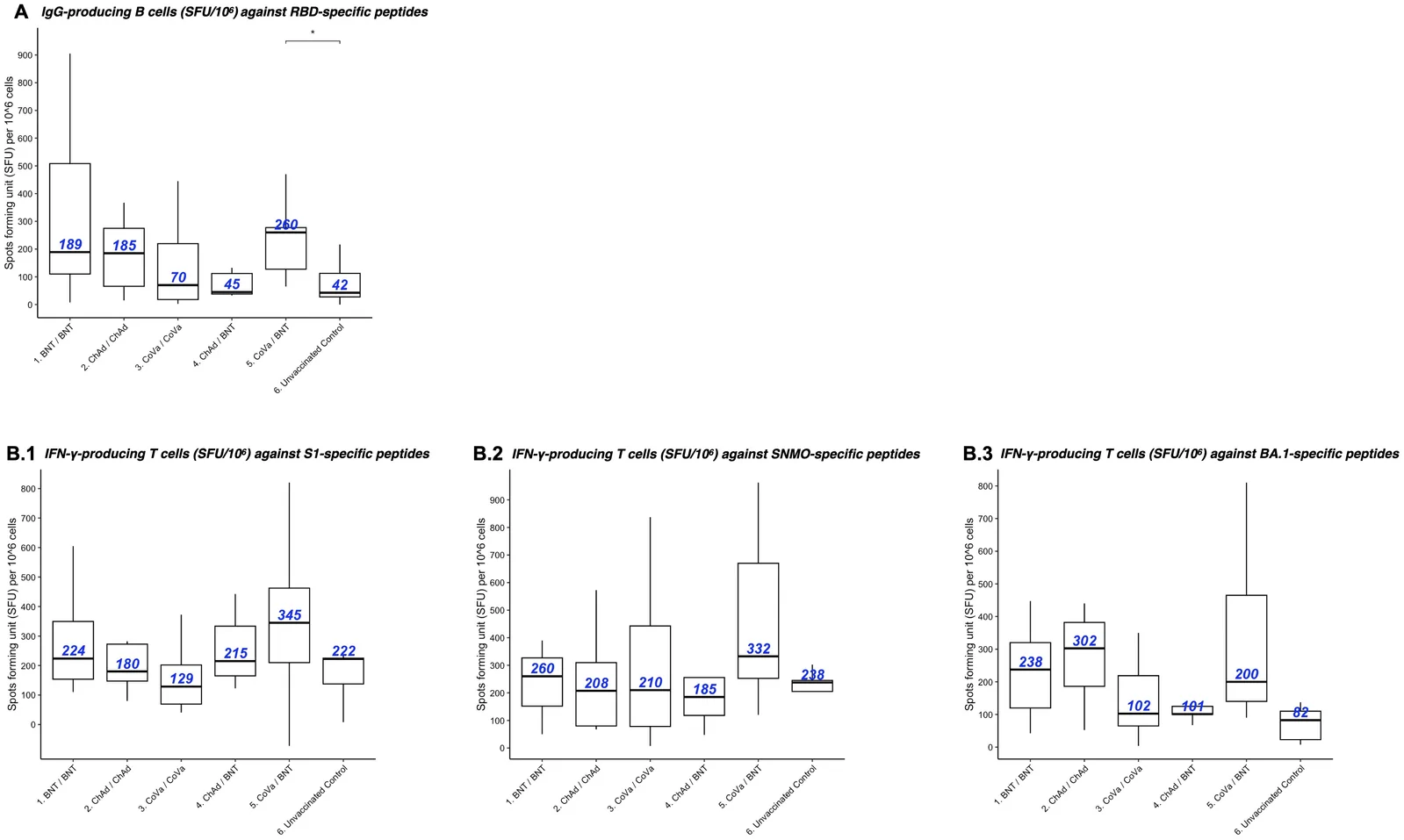
ELISpot analysis of RBD-specific IgG-producing B cells and IFN-γ-producing T cells across different COVID-19 vaccination regimens at the 12-month follow-up. **(A)** Frequencies of RBD-specific IgG-producing B cells following stimulation with R848 and IL-2. **(B)** Frequencies of IFN-γ-producing T cells following stimulation with SARS-CoV-2 S1, SNMO, and Omicron BA.1 peptide pools. Data are presented as spot-forming units per million cells (SFU/10^6^ cells). Box plots represent median SFU values with interquartile ranges. Mann-Whitney U test was performed to measure the differences in SFU between the study groups. Significant differences are marked as *p < 0.05. BNT, BNT162b2 vaccine; ChAd, ChAdOx1 nCoV-19 vaccine; CoVa, CoronaVac vaccine; “/” separates the two-dose primary vaccination series and a booster dose in chronological order.

To assess the functional capacity of vaccine-elicited B and T cell responses, ELISpot assays were performed on PBMCs collected at the 12-month follow-up. A statistically significant increase in RBD-specific IgG-producing B cells was observed in the heterologous CoronaVac/BNT162b2 vaccination regimen (median = 260 SFU/10^6^ cells) compared to the unvaccinated control group (median = 42 SFU/10^6^ cells, p < 0.05) (**Figure 3A**). In contrast, no statistically significant differences were detected in IFN-γ-producing T cells across the vaccination regimens when stimulated with S1-specific (median range = 129–345 SFU/10^6^ cells), SNMO-specific (median range = 185–332 SFU/10^6^ cells), or BA.1-specific (median range = 82–302 SFU/10^6^ cells) peptide pools (**Figure 3B**). However, the heterologous CoronaVac/BNT162b2 vaccination regimen exhibited the highest median SFU/10^6^ cells against S1-specific and SNMO-specific SARS-CoV-2 peptides (**Figure 3B.1**).

**Figure 4:**
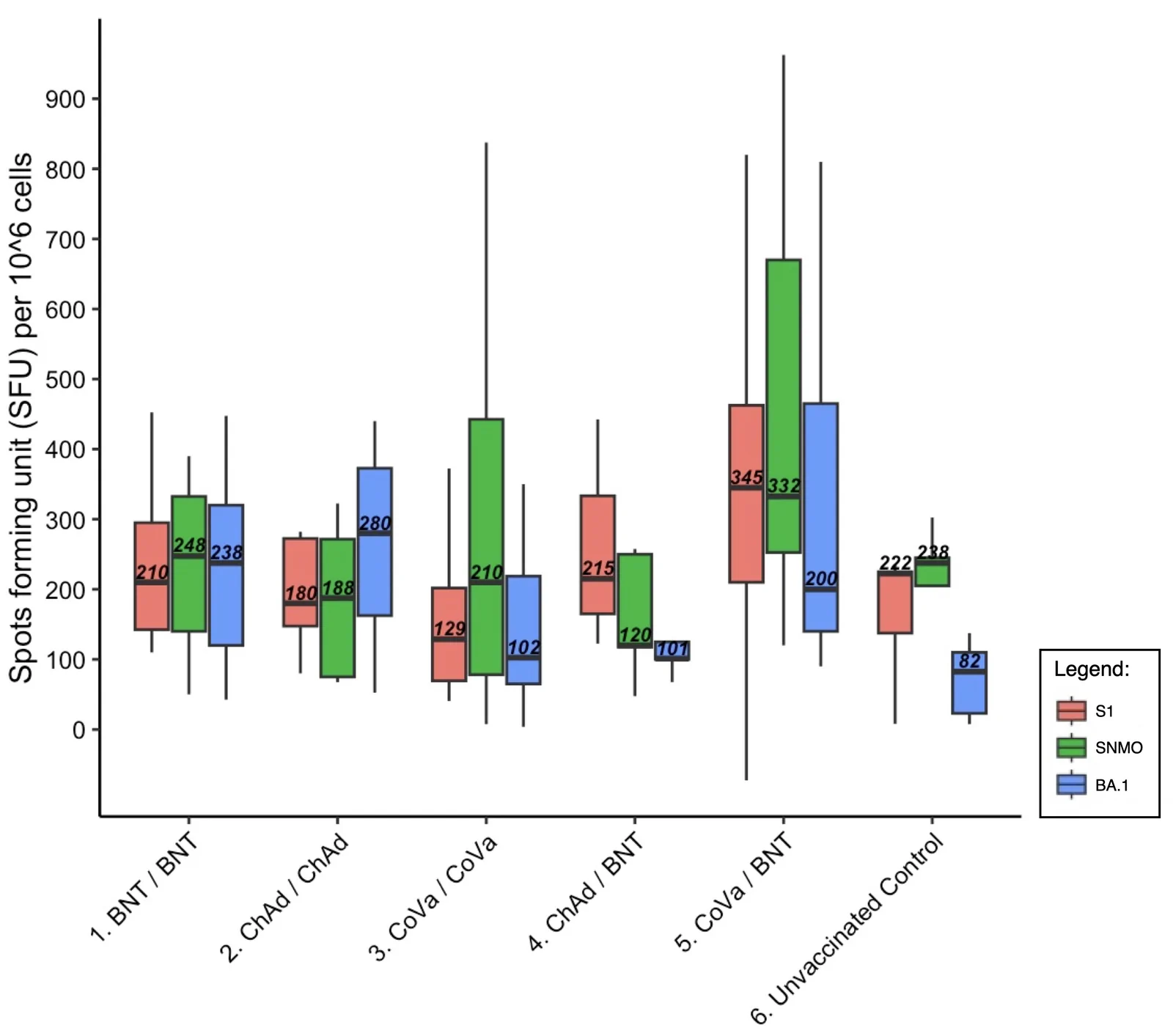
Comparison of IFN-γ-producing T cell responses against SARS-CoV-2 S1, SNMO, and Omicron BA.1 peptide pools across different COVID-19 vaccination regimens at the 12-month follow-up. Data are presented as spot-forming units per million cells (SFU/10^6^ cells). Box plots represent median SFU values with interquartile ranges. Mann-Whitney U test was performed to measure the differences in SFU between the study groups. *Abbreviations:* BNT, BNT162b2 vaccine; ChAd, ChAdOx1 nCoV-19 vaccine; CoVa, CoronaVac vaccine; “/” separates the two-dose primary vaccination series and a booster dose in chronological order.

ELISpot assays revealed that the unvaccinated control group consistently exhibited the lowest median IFN-γ-producing T cell responses across all three peptide pools (S1, SNMO, and BA.1), with median values of 222, 238, and 82 SFU/10^6^ cells, respectively. In contrast, the heterologous CoronaVac/BNT162b2 vaccination regimen showed higher median SFU, with values of 345, 332, and 200 SFU/10^6^ cells for S1, SNMO, and BA.1, respectively (**Figure 4**). However, these differences did not reach statistical significance between the study groups.

### 3.4 SARS-CoV-2 S1-specific IgG levels in PBMCs supernatant before and after stimulation

**Figure 5:**
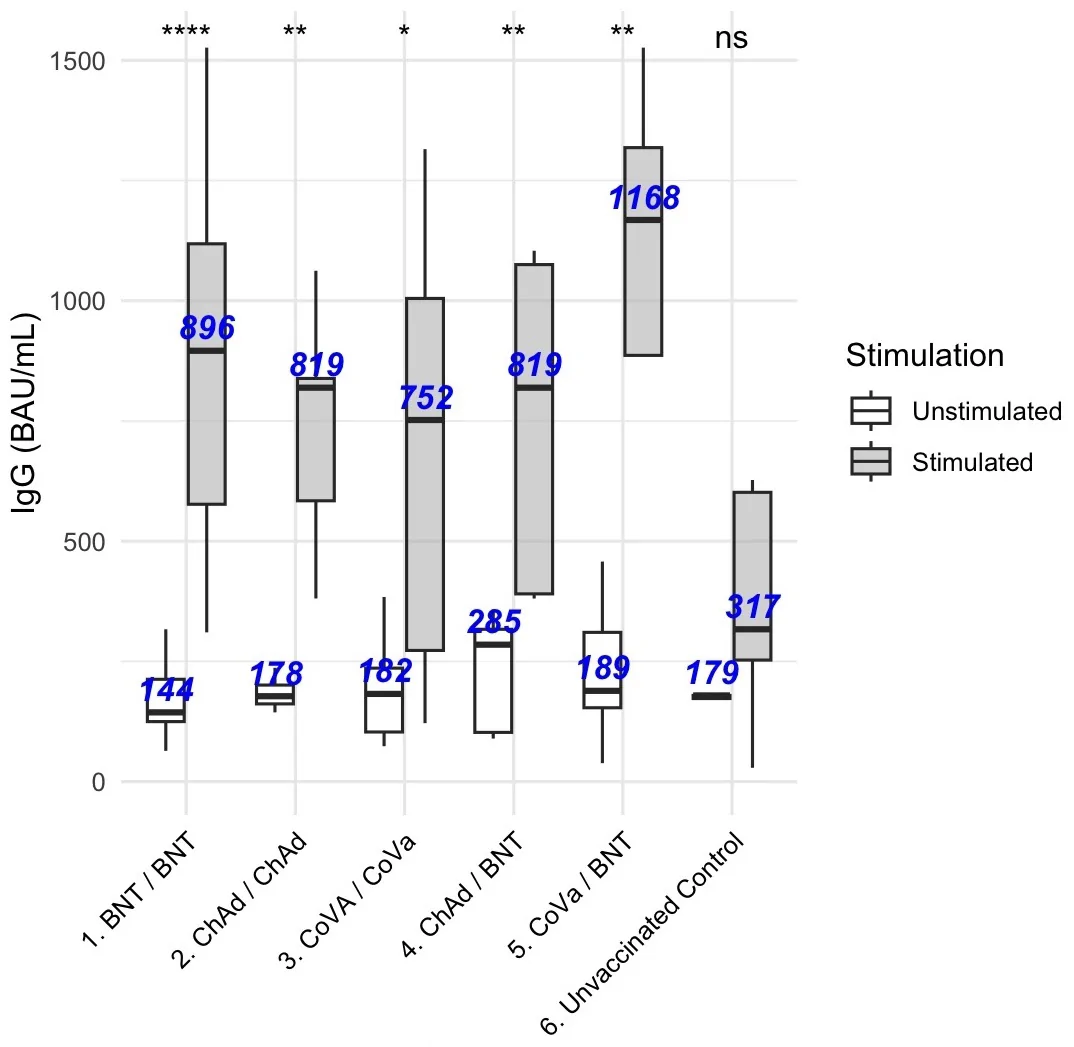
SARS-CoV-2 S1-specific IgG levels in PBMCs supernatants before and after stimulation across different vaccination regimens at the 12-month follow-up. Boxplots represent IgG (BAU/mL) levels in unstimulated (white) and stimulated (gray) conditions. Blue numbers indicate median IgG levels for each condition. Significant differences are marked as *p < 0.05, **p < 0.01, ***p < 0.001, ****p < 0.0001. “ns” indicates no significant difference. BNT, BNT162b2 vaccine; ChAd, ChAdOx1 nCoV-19 vaccine; CoVa, CoronaVac vaccine; “/” separates the two-dose primary vaccination series and a booster dose in chronological order.

The analysis of SARS-CoV-2 S1-specific IgG levels in PBMCs supernatants across different vaccination regimen groups revealed significant differences in antibody recall responses following stimulation (**Figure 5**). Overall, stimulated PBMCs supernatant showed an increase in SARS-CoV-2 S1-specific IgG levels across all vaccinated groups. Participants receiving heterologous vaccination regimens (ChAdOX1 nCoV-19/BNT162b2 and CoronaVac/BNT162b2) and homologous BNT162b2 vaccination regimen exhibited the highest IgG responses, with heterologous CoronaVac/BNT162b2 showing the highest median stimulated IgG levels (1168 BAU/mL). The unvaccinated individuals showed minimal SARS-CoV-2 S1-specific IgG levels, with no significant increase upon stimulation.

### 3.5 Principal component analysis of cytokine, chemokine, and growth factor responses in PBMCs supernatant before and after stimulation

PCA was performed as an exploratory visualization to summarize combined cytokine, chemokine, and growth factor levels before and after stimulation. Given the high dimensionality of the dataset and biological overlap between vaccination regimens, PCA clusters were not expected to be fully separable.

**Figure 6:**
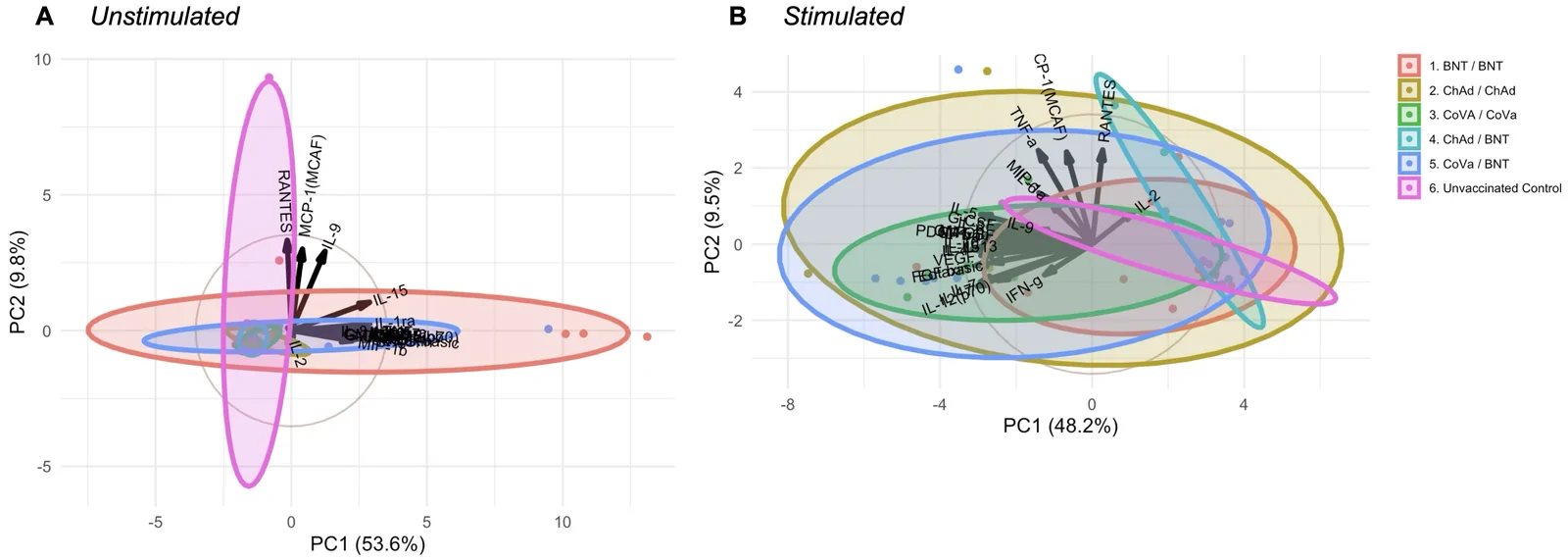
Principal Component Analysis (PCA) of cytokine, chemokine, and growth factor responses across different COVID-19 vaccination regimens at the 12-month follow-up. PCA plots are shown for **(A)** Unstimulated, and **(B)** Stimulated conditions. Each point represents an individual participant, while ellipses represent the 95% confidence interval for each study group. The principal component axes indicate the percentage of variance explained. Arrows represent cytokines, chemokines, and growth factors contributing to variance within the principal component space. BNT, BNT162b2 vaccine; ChAd, ChAdOx1 nCoV-19 vaccine; CoVa, CoronaVac vaccine; “/” separates the two-dose primary vaccination series and a booster dose in chronological order.

The PCA biplot illustrated the cytokine, chemokine, and growth factor response before PBMCs stimulation, with the first principal component (PC1) explaining 53.6% of the variance and the second principal component (PC2) accounting for 9.8% (**Figure 6A**). The cytokines driving separation include RANTES, MCP-1 (MCAF), IL-9, and IL-15. The homologous BNT162b2 vaccination regimen (red) exhibited a broad distribution along PC1, showing a more pronounced cytokine response with higher variation. In contrast, the heterologous CoronaVac/BNT162b2 vaccination regimen (blue) exhibited a compact and clustered pattern, showing a more consistent cytokine response. The unvaccinated control group (pink) formed a distinct cluster, primarily driven by RANTES and MCP-1. The homologous ChAdOx1 nCoV-19 and heterologous ChAdOX1 nCoV-19/BNT162b2 vaccination regimens (green and cyan) remained centered, showing a more compact distribution.

After PBMCs stimulation, the PC1 showed 48.2% of the variation, while the PC2 accounted for 9.5%. RANTES, MCP-1 (MCAF), TNF-α, IFN-γ, and IL-9 drove the separation along these axes, influencing group clustering (**Figure 6B**). Among the study groups, homologous BNT162b2 and heterologous CoronaVac/BNT162b2 showed similar clustering patterns with narrower distributions along PC1. Conversely, homologous ChAdOx1 nCoV-19 and heterologous ChAdOX1 nCoV-19/BNT162b2 exhibited a broader distribution along PC1. The homologous CoronaVac vaccination regimen was positioned more negatively on PC1, showing a distinct cytokine response pattern. The unvaccinated control group clustered near the center with a smaller spread compared with the vaccinated study groups.

### 3.6 Magnitude changes in cytokine, chemokine, and growth factor expression in PBMC supernatant before and after stimulation

**Figure 7:**
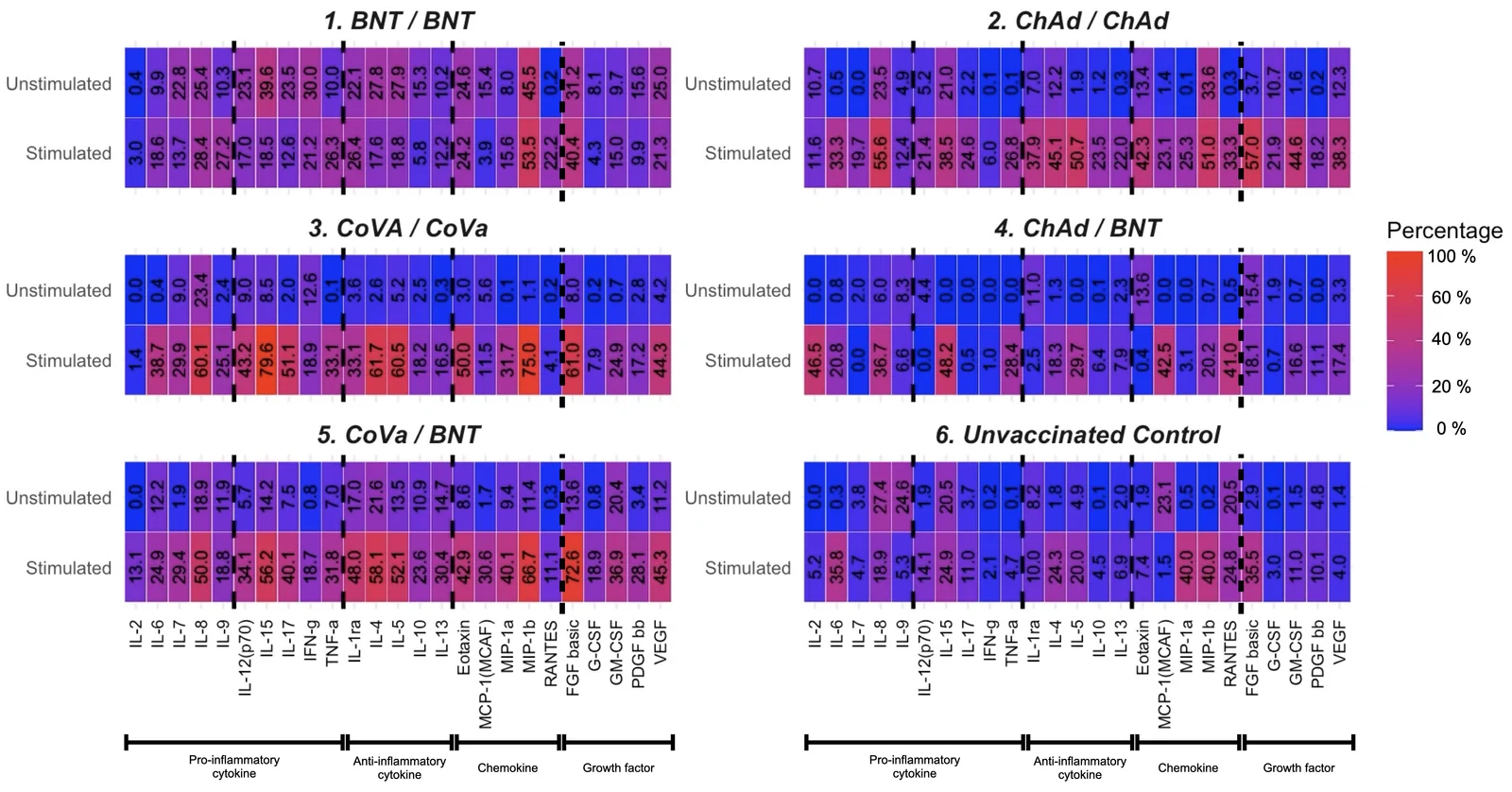
Heatmap representation of cytokine, chemokine, and growth factor levels under unstimulated and stimulated conditions across different COVID-19 vaccination regimens at the 12-month follow-up. Colors correspond to relative analyte levels, as indicated by the scale bar (red = higher values; blue = lower values). Unstimulated and stimulated conditions are presented for visual comparison of response patterns following stimulation. BNT, BNT162b2 vaccine; ChAd, ChAdOx1 nCoV-19 vaccine; CoVa, CoronaVac vaccine; “/” separates the two-dose primary vaccination series and a booster dose in chronological order.

Heatmap analysis of cytokine, chemokine, and growth factor levels revealed distinct patterns of expression in both unstimulated and stimulated conditions (**Figure 7**). In general, stimulation was associated with increased pro-inflammatory cytokines such as IL-6 and CXCL8 (also known as IL-8) across all vaccination regimens, with the homologous CoronaVac vaccination regimen exhibiting comparatively higher levels. The anti-inflammatory cytokine IL-1ra also showed a trend toward increased expression upon stimulation. The unvaccinated control group generally exhibited lower levels of cytokine production compared to the vaccinated groups (**Supplementary Figure 4**).

### 3.7 Significant differences of cytokine, chemokine, and growth factor expression

**Figure 8:**
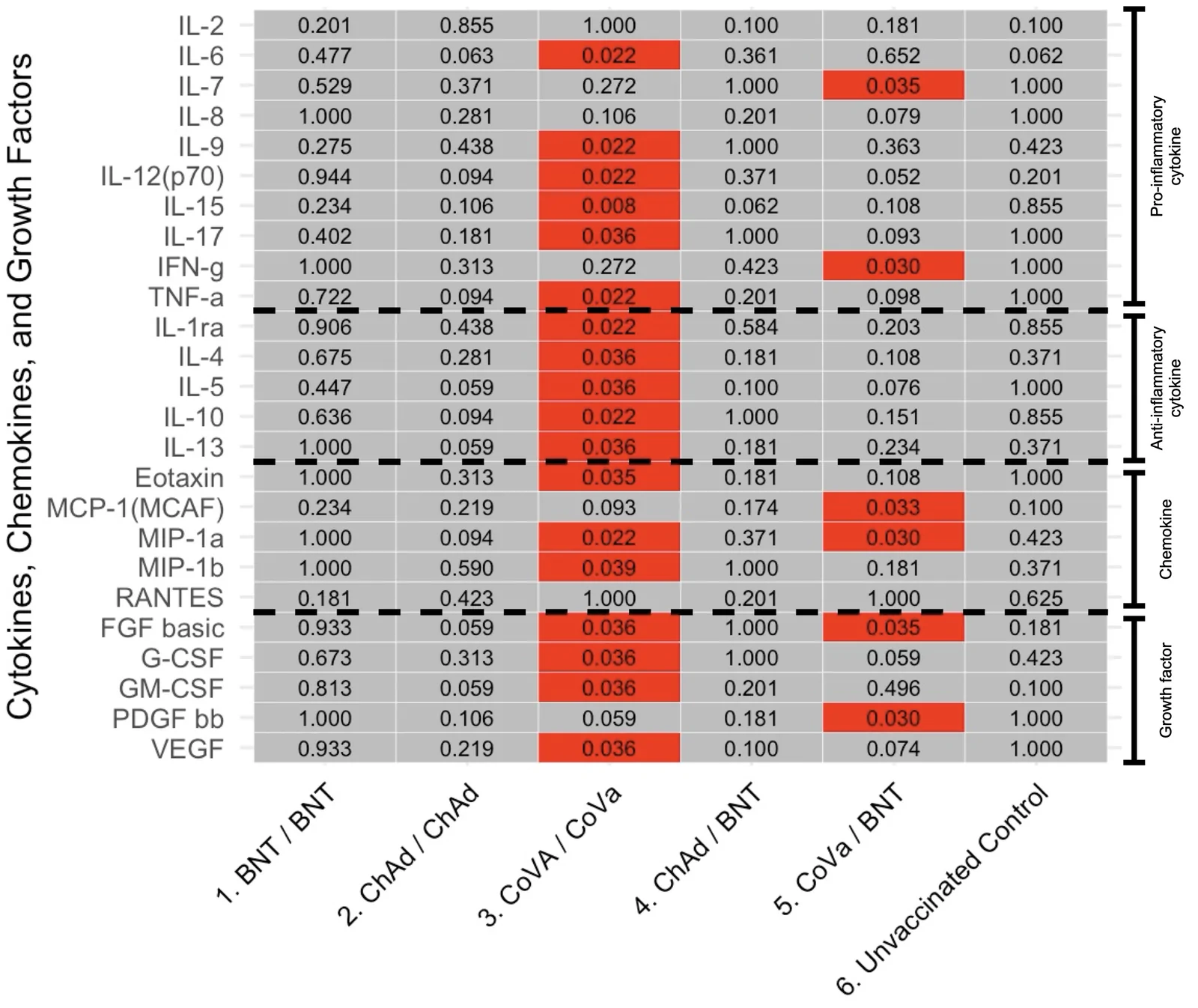
Summary matrix of cytokine, chemokine, and growth factor responses following stimulation across different COVID-19 vaccination regimens at the 12-month follow-up. Statistically significant differences between stimulated and unstimulated conditions are highlighted in red (*p* < 0.05). Values within each tile represent corresponding the *p*-values. BNT, BNT162b2 vaccine; ChAd, ChAdOx1 nCoV-19 vaccine; CoVa, CoronaVac vaccine; “/” separates the two-dose primary vaccination series and a booster dose in chronological order.

The homologous CoronaVac vaccination regimen exhibited the broadest significant response profile, with increased levels of IL-6, IL-9, IL-12(p70), IL-15, IL-17, TNF-α, IL-1ra, IL-4, IL-5, IL-10, IL-13, Eotaxin (CCL11), MIP-1α, MIP-1β, FGF basic, G-CSF, GM-CSF, and VEGF (*p* < 0.05) (**Figure 8**). The heterologous CoronaVac/BNT162b2 vaccination regimen also demonstrated significant increases in IL-7, IFN-γ, MCP-1(MCAF), MIP-1α, FGF basic, and PDGF bb (*p* < 0.05). In contrast, the homologous BNT162b2, homologous ChAdOX1 nCoV-19, heterologous ChAdOX1 nCoV-19/BNT162b2, and the unvaccinated control groups, showed no significant differences between stimulated and unstimulated conditions; however, interpretation is limited by the very small sample size (n = 5), which substantially reduced statistical power.

### 3.8 Correlation between cytokine, chemokine, and growth factor responses with B cells

**Figure 9:**
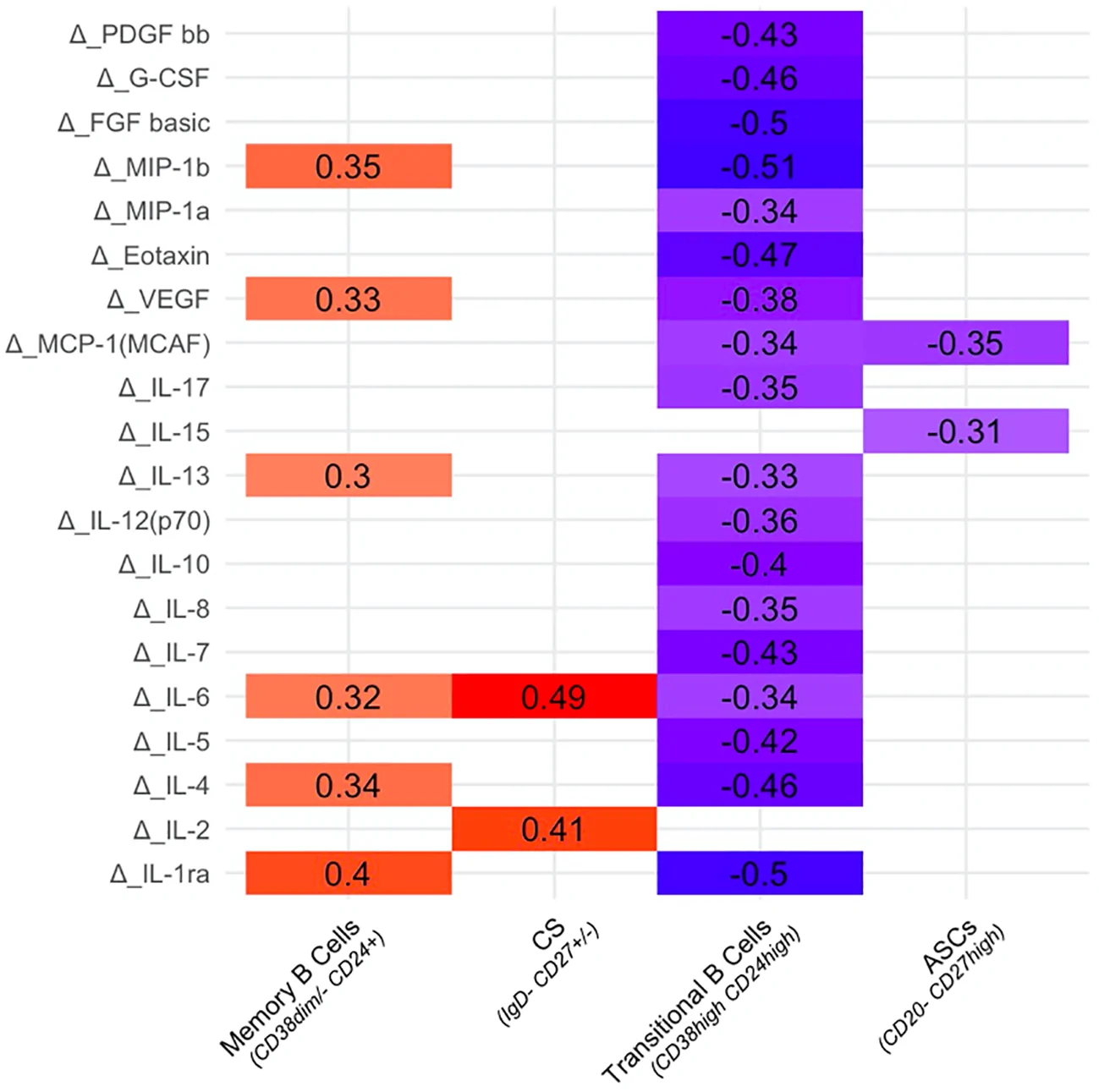
Correlations between cytokine, chemokine, growth factor levels and B cell subpopulation frequencies at the 12-month follow-up. Only statistically significant Spearman correlations (*p* < 0.05) are displayed. Correlation coefficients are represented according to the scale shown, where blue tiles indicate negative correlations and red tiles indicate positive correlations. Increasing color intensity reflects stronger correlation strength. Δ (delta) denotes fold change in cytokine, chemokine, and growth factor levels following stimulation. CS, class-switched; ASCs, antibody-secreting cells.

Spearman correlations were calculated to examine the relationship between fold changes (Δ) in cytokine, chemokine, and growth factor levels and the frequencies of B cell subpopulations (**Figure 9**). A consistent pattern of negative correlations was observed between transitional B cells and most cytokine fold changes, whereby higher cytokine fold changes corresponded with a lower proportion of transitional B cells. A positive correlation was noted for the fold change in IL-6 level with frequencies of both class-switched memory and total memory B cells, while these subsets also displayed weak correlations with fold changes in the level of other cytokines.

## 4 Discussion

During the COVID-19 pandemic, multiple vaccine platforms were deployed. While many regions relied predominantly on either mRNA or inactivated viral vaccines, Malaysia was a unique setting in which multiple COVID-19 vaccine platforms, including mRNA (BNT162b2), adenoviral vector (ChAdOx1 nCoV-19), and inactivated virus (CoronaVac), were deployed concurrently as part of the national immunization program (12). This diversity of vaccination combinations across the population provided a valuable opportunity to examine how different platforms with variable antigenic and immunogenic compositions shape long-term adaptive immune memory. Understanding vaccine-induced immunological differences is essential for guiding booster strategies and informing future vaccine design, particularly in settings where mixed-platform vaccination could be implemented. Notably, significant variability in clinical outcomes has been reported across different vaccine platforms in preventing severe SARS-CoV-2 infection (21–25). As vaccine-elicited serum IgG and neutralizing antibody titres naturally declines over time (26), the contribution of memory B and T cells becomes increasingly important for sustaining long-term protection by enabling rapid antibody production and cellular recall responses upon antigen re-exposure (11). In this study, analysis at 12-month follow-up revealed distinct immunological signatures shaped by homologous and heterologous vaccination strategies. A key finding was the significantly higher frequencies of Spike++ memory B cells in individuals who received a homologous two-dose BNT162b2 primary vaccination series followed by a BNT162b2 booster dose, despite comparable RBD^+^ memory B cell responses across the different vaccination regimens. These findings suggest that BNT162b2 may induce a more robust spike protein-specific memory, targeting epitopes beyond the RBD. Such broader targeting of the spike protein could enhance protection against variants by eliciting antibodies against conserved, non-RBD epitopes, potentially mitigating the impact of RBD mutations on antibody binding and neutralization (27).

Repeated exposure to the full-length spike protein through the mRNA platform may be associated with broader expansion of memory B cells by targeting multiple regions of the spike protein (28–30). Additionally, the specific adjuvants and delivery mechanisms of the BNT162b2 vaccine may contribute to this enhanced B cell response. No significant differences were observed in other B cell subpopulations including mature, memory, class-switched, non-class-switched, naïve, transitional, plasmablasts, and antibody-secreting cells across different vaccination regimen of BNT162b2, ChAdOx1 nCoV-19, and CoronaVac. These findings suggest comparable long-term maintenance of B cell subpopulations across COVID-19 vaccination regimens.

The increased RBD-specific IgG-producing B cells in the heterologous CoronaVac/BNT162b2 regimen, as measured by ELISpot, support previous observations that heterologous vaccination may enhance long-term immunity (31). The increase in SARS-CoV-2 S1-specific IgG levels after a two-dose CoronaVac primary vaccination series followed by a BNT162b2 booster suggested improved B cell recall, likely due to broader immune imprinting or enhanced affinity maturation (31). One possible mechanism is that inactivated virus vaccines like CoronaVac prime the immune system with diverse viral antigens (21, 23), while an mRNA booster strengthens the spike-specific response, increasing RBD-specific IgG production (6, 32). This heterologous regimen has previously been associated with stronger and more durable antibody responses compared to homologous regimens (5, 33). Consistent with our findings, heterologous CoronaVac/BNT162b2 vaccination has also been associated with increased RBD-binding memory B cells, enhanced Spike-specific IFN-γ-producing T-cell responses, and superior neutralizing antibody responses compared with homologous CoronaVac vaccination (5). The inactivated virus vaccine may contribute to broader cross-reactive antibodies, allowing the mRNA booster to refine the Spike-specific response.

IFN-γ-producing T cell responses varied considerably across individuals and tended to be the lowest in the unvaccinated group, underscoring the role of vaccination in priming T cell immunity. Strong T cell responses are essential for viral clearance and adaptive immunity, with potential cross-protection against SARS-CoV-2 variants (34). The heterologous CoronaVac/BNT162b2 regimen showed the highest IFN-γ recall response, aligning with increased RBD-specific IgG-producing B cells, suggesting that stronger RBD-specific memory B cell responses may be associated with enhanced IFN-γ recall responses upon SARS-CoV-2 exposure. Such a response may be consistent with enhanced CD4^+^ T cell-associated immunity and could provide cross-protection against emerging SARS-CoV-2 variants (4, 34). While direct regulation of IFN-γ by these B cells is not well-documented, their presence is consistent with enhanced cellular immune activation (35, 36). IFN-γ plays a key role in vaccine-induced immunity (36), and its co-production with IL-10 has been linked to effective viral control and asymptomatic infection (35). The association between RBD-specific memory B cells and IFN-γ-producing T cells may reflect coordinated immune activation that contributes to improved SARS-CoV-2 immune control following antigen re-exposure.

The heterogeneity of cytokine, chemokine, and growth factor profiles observed across the different vaccination regimens highlighted the influence of the vaccine platform on the innate and adaptive immune responses. The broader cytokine upregulation observed in the homologous CoronaVac vaccination regimen aligns with the diverse antigenic exposure associated with inactivated vaccines (37), whereas the more specific response seen in mRNA-based regimens may relate to their Spike-directed immunogenicity (6).

In the unstimulated condition, cytokine expression remained relatively uniform. At the 12-month follow-up, recipients of the homologous BNT162b2 vaccine exhibited a broad cytokine profile, consistent with enhanced immune recall. In contrast, the unvaccinated group showed a distinct cytokine pattern, characterized by elevated RANTES and MCP-1, suggesting a unique immune activation pathway which may reflect prior natural infection rather than vaccine-induced immune responses. RANTES and MCP-1 play roles in immune cell recruitment and activation (38). Following stimulation, vaccinated individuals demonstrated a more diverse and robust cytokine response profile compared to unvaccinated individuals, consistent with studies showing that vaccination significantly enhances cellular immunity (39), highlighting the enhanced recall capacity of vaccine-induced immunity.

The cytokines, chemokines, and growth factors evaluated in this study are involved in multiple immune pathways, including inflammatory responses, Th1 and Th2 immunity, immune regulation, immune cell recruitment, and tissue repair (10, 35, 40–42). The present study showed that homologous CoronaVac and BNT162b2-boosted CoronaVac regimens elicit broad cytokine activation. Homologous CoronaVac significantly upregulated IL-6, IL-9, IL-12(p70), IL-15, IL-17, TNF-α, IL-1ra, IL-4, IL-5, IL-10, IL-13, Eotaxin (CCL11), MIP-1α, MIP-1β, FGF basic, G-CSF, GM-CSF, and VEGF, consistent with previous studies (37). In contrast, CoronaVac-primed and BNT162b2-boosted individuals showed significant upregulation of IL-7, IFN-γ, MCP-1, MIP-1α, FGF basic, and PDGF bb, aligning with other findings (10, 43). These results highlight the priming vaccine’s role in shaping memory B cell responses, with whole-virus vaccines like CoronaVac potentially leading to a more diverse cytokine profile (31, 37, 38). While this broader cytokine response may reflect a more polyfunctional memory B cell repertoire, further research is needed to determine its impact on clinical protection (10, 37).

Correlation analysis of fold changes in most cytokines, chemokines, and growth factors exhibited a consistent pattern of negative correlations with transitional B cells. Likely increased cytokine production is associated with a decrease in the proportion of these immature B cells (37, 44, 45). Conversely, IL-6 levels showed a positive correlation with class-switched memory B cells and total memory B cells, consistent with its role in promoting B cell differentiation and antibody production via STAT3 signaling pathways (37, 46). These associations support a role for IL-6–mediated signaling in shaping B cell maturation toward memory phenotypes and may reflect the influence of priming antigenic exposures on subsequent immune programming. Inactivated virus vaccines, which present a broader array of viral antigens, may provide a more diverse initial antigenic stimulus for improved subsequent recall responses when followed by an mRNA booster (5, 33). This pattern may reflect cytokine-associated B cell maturation pathways following CoronaVac priming, where IL-6 may contribute to the transition of transitional B cells into mature memory subsets and support long-term immune memory maintenance (44, 45). The negative correlations between MCP-1 (MCAF) and antibody-secreting cells (ASCs) may reflect regulatory immune interactions during peak inflammatory responses (37, 47). These findings highlight a coordinated interplay between cytokine dynamics and B cell maturation, where pro-differentiation signals (e.g., IL-6) and regulatory chemokines (e.g., MCP-1) may influence the magnitude and durability of vaccine-induced immunity (37, 44, 45, 47). The broader cytokine activation observed following CoronaVac-containing regimens may also be interpreted within the broader framework of vaccine-induced immune programming, including the emerging concept of trained immunity. However, as the present study focused on adaptive immune memory and did not assess innate immune cell function, no conclusions regarding trained immunity can be drawn. Future studies integrating innate and adaptive immune profiling will be valuable to determine how heterologous vaccination strategies differentially shape trained immunity and long-term adaptive immune memory.

Several limitations in the study warrant consideration. The restricted availability of PBMCs precluded detailed flow-cytometric characterization of T-cell subsets, and IFN-γ–producing T-cell responses demonstrated substantial inter-individual variability without achieving statistical significance. Clinical endpoints, including progression to severe disease or hospitalization, were not captured, and neither innate immune responses nor antibody functional properties were assessed. An additional limitation is the potential confounding effect of demographic differences between vaccination groups. Participants in the heterologous CoronaVac/BNT162b2 group were exclusively female and generally younger than those in several other groups. Therefore, the enhanced immune responses observed in this group may have been influenced, at least in part, by these demographic characteristics in addition to the vaccination regimen itself. Larger studies with balanced demographic distributions and multivariable adjustment will be required to further disentangle these effects. The cross-sectional design, with PBMCs collection performed at the 12-month follow-up after completion of the primary vaccination series, limits inferences regarding temporal trajectories of cellular immunity and the durability of responses beyond this interval. In addition, participants were not systematically screened for interim SARS-CoV-2 infection; therefore, undocumented infections and hybrid immunity could not be excluded. Accordingly, the observed immune responses likely reflect real-world immune status rather than vaccine-induced immunity alone. The observational nature of the study also restricted causal interpretation, and the modest sample size reduces statistical power. In particular, the small number of participants in the unvaccinated control group limited the statistical power of within-group paired analyses, and the absence of statistically significant changes following stimulation should therefore be interpreted with caution. Furthermore, multiple statistical comparisons were performed across numerous immunological endpoints. As this study was exploratory in nature, formal adjustment for multiple testing was not applied. Consequently, some statistically significant findings should be interpreted with caution and warrant validation in larger independent cohorts. Future investigations incorporating clinical outcomes, expanded immunological profiling, and larger longitudinal cohorts will be essential to more precisely define the durability and determinants of long-term immunity under real-world conditions.

## 5 Conclusion

The study highlighted distinct long-term immunological profiles associated with different COVID-19 vaccination strategies, particularly the homologous BNT162b2 and heterologous CoronaVac-primed, BNT162b2-boosted regimens. Homologous BNT162b2 vaccination was associated with higher frequencies of Spike-specific memory B cells, while the heterologous CoronaVac/BNT162b2 regimen demonstrated enhanced RBD-specific recall responses and broader cytokine activation patterns. Collectively, these findings advance understanding of how distinct antigenic exposures shape adaptive immune responses and highlight the potential advantages of specific vaccination strategies in sustaining long-term immune memory against SARS-CoV-2 under real-world conditions at the 12-month follow-up. Continued investigation into the mechanisms driving these divergent immune profiles will be critical for guiding the design of next-generation vaccines and optimizing immunization strategies, particularly as viral variants continue to evolve.

## Data Availability

The raw data supporting the conclusions of this article will be made available by the authors, without undue reservation.

## Author disclaimer

HN, RH, AL are military service members. This work was prepared as part of their official duties. Title 17, U.S.C., §105 provides that copyright protection under this title is not available for any work of the U.S. Government. Title 17, U.S.C., §101 defines a U.S. Government work as a work prepared by a military Service member or employee of the U.S. Government as part of that person’s official duties. The views expressed in the article are those of the authors and do not necessarily express the official policy and position of the U.S. Navy, the Department of Defense, the U.S. government, or any of the other institutions affiliated with any of the authors.

## Supplementary material

Supplementary Figure 1 Gating strategies to identify B cell subpopulations. representative plots show that the live cells were identified based on 7-AAD staining and singlets cells identified by FSC-A vs. FSC-H, followed by FSC-A vs. SSC-A for lymphocyte population. B cell subpopulations were identified based on the expression of surface markers such as mature B cell lineage (CD19^+^ CD20^+^), transitional B cell (CD38^dim/-^ CD24^+^), antibody-secreting cell (CD38^+^ CD24^-^ CD27^+^ CD20^-^), CD27 and IgD for atypical memory B cells, memory B cells, innate-like memory B cells, naïve B cells. Spike and RBD-specific SARS-CoV-2 markers, including BB515 Streptavidin BV421 Streptavidin for double Spike^+^, BB515 Streptavidin BV421 Streptavidin for double Spike^+^ with Spike RBD^+^, and IgM with IgG were used to distinguish the primary and secondary response.

Supplementary Figure 2 Overview of ELISpot workflow. Figure created using BioRender.com.

Supplementary Figure 3 Representative ELISpot images of PBMCs responses against SARS-CoV-2 peptides at the 12 months following COVID-19 vaccinations. Images are representative of data generated in this study. ELISpot assays are shown for **(A)** R848 peptides which target RBD-specific cells, and **(B)** peptide pools which target S1, SNMO, and BA.1 Omicron. Unstimulated cells served as negative controls, while nonspecific stimulants were used as positive controls to assess cell viability. The spots were quantified using an ImmunoSpot analyzer and presented in Spot Forming Units (SFU) per million cells. RPMI, Roswell Park Memorial Institute media; FBS, Fetal Bovine Serum; SFU, Spot-Forming Units; PBMCs, peripheral blood mononuclear cells.

Supplementary Figure 4 Magnitude changes in cytokine, chemokine, and growth factor expression before and after PBMCs stimulation. Boxplots represent the levels in unstimulated (blue) and stimulated (brick red) conditions. Median levels for each condition were shown in the boxplot. Statistical significance of differences between the study groups is denoted by asterisks (*p < 0.05, **p < 0.01, ****p < 0.0001, ns = not significant). BNT, BNT162b2 vaccine; ChAd, ChAdOx1 nCoV-19 vaccine; CoVa, CoronaVac vaccine; “/” separates the two-dose primary vaccination series and a booster dose in chronological order.
